# Comparison of Cannabinoid CB_1_ Receptor Binding in Adolescent and Adult Rats: A Positron Emission Tomography Study Using [^18^F]MK-9470

**DOI:** 10.1155/2011/548123

**Published:** 2011-12-11

**Authors:** Mathieu Verdurand, Vu Nguyen, Daniela Stark, David Zahra, Marie-Claude Gregoire, Ivan Greguric, Katerina Zavitsanou

**Affiliations:** ^1^Schizophrenia Research Institute, Sydney, Australia; ^2^ANSTO LifeSciences, ANSTO, PMB 1 Menai, Sydney, Australia; ^3^School of Psychiatry, Faculty of Medicine, University of New South Wales, Sydney, NSW, Australia; ^4^Schizophrenia Research Laboratory, Neuroscience Research Australia, Randwick, NSW, Australia

## Abstract

Despite the important role of cannabinoid CB_1_ receptors (CB_1_R) in brain development, little is known about their status during adolescence, a critical period for both the development of psychosis and for initiation to substance abuse. In the present study, we assessed the ontogeny of CB_1_R in adolescent and adult rats *in vivo* using positron emission tomography with [^18^F]MK-9470. Analysis of covariance (ANCOVA) to control for body weight that would potentially influence [^18^F]MK-9470 values between the two groups revealed a main effect of age (F(1,109)=5.0, *P* = 0.02) on [^18^F]MK-9470 absolute binding (calculated as percentage of injected dose) with adult estimated marginal means being higher compared to adolescents amongst 11 brain regions. This finding was confirmed using *in vitro* autoradiography with [^3^H]CP55,940 (F(10,99)=140.1, *P* < 0.0001). This ontogenetic pattern, suggesting increase of CB_1_R during the transition from adolescence to adulthood, is the opposite of most other neuroreceptor systems undergoing pruning during this period.

## 1. Introduction

The endocannabinoid system is a lipid signalling system [1] that appeared early in evolution [2]. It consists of at least two G-protein-coupled cannabinoid receptors CB_1_ and CB_2_ (CB_1_R and CB_2_R) [3], their intrinsic ligands (endocannabinoids) such as N-arachidonoyl ethanolamine (anandamide, AEA) [4] and 2-arachidonoyl glycerol (2-AG) [5], and their associated proteins involved in synthesis, transport, and degradation [6].

The CB_1_R, which mediates the psychoactive effects of marijuana, is widely expressed and is considered one of the most abundant G-protein-coupled receptors in the brain. In the central nervous system, endocannabinoids are released from postsynaptic sites and, by activation of the presynaptically located CB_1_R [7], inhibit the release of several neurotransmitters such as GABA, glutamate [8], dopamine, and acetylcholine [9]. *In vitro* immunohistochemical [10] and autoradiography [11] studies in rats have shown that the CB_1_R is highly expressed in the basal ganglia (lateral caudate-putamen, globus pallidus, entopeduncular nucleus, and substantia nigra pars reticulata), cerebellum (molecular layer), and hippocampus (CA1, CA3, and dentate gyrus molecular layer). Moderate levels are found throughout the cortical regions, whereas low levels are observed in the brainstem (midbrain, pons) and spinal cord.

The CB_1_R has been shown to be involved in various physiological functions like nociception [12], control of movement [13], memory [14], neuroendocrine regulation [15], brain development, and maturation [16, 17]. Biochemical and functional alterations of CB_1_R have been shown to be implicated in the pathophysiology of distinct neurological and psychiatric disorders [18] including schizophrenia [19–21]. It is known that cannabis and its derivatives can trigger psychotic-like symptoms in normal individuals [22], and numerous epidemiological studies have demonstrated that consuming cannabis during adolescence (particularly early adolescence) constitutes a risk factor for schizophrenia onset later in life [23–25].

Adolescence is a critical developmental period during the transition from childhood to adulthood. The ages associated with adolescence are commonly considered in humans to be approximately 12 to 20–25 years of age and postnatal day (PND) 28–55 in rodents [26]. The adolescent brain undergoes both progressive and regressive changes providing the biological basis for the unique adolescent behaviors and their associated changes during maturation to adulthood. At the cellular level, these changes correspond to the marked overproduction of axon and synapses in early puberty and rapid pruning in late adolescence [27]. To date, most developmental studies of the cannabinoid system [28–31] have focused on the embryonic and early postnatal stages. *In vitro* autoradiographic studies have reported a fivefold increase in CB_1_R density in the brain during postnatal development [32]. CB_1_R capacity in the striatum was doubled between PND 14 and 21. Significant increases in CB_1_R density appeared regionally in the developing brain until PND 21 [32] or PND 30 [33], and the maximum adult level was reached at PND 60 [32]. In contrast, Rodriguez de Fonseca et al. [33] reported slight decreases in binding between PND 30 and 40 and adulthood (PND 70).

Recently, the development of new efficient radiotracers has enabled the study of CB_1_R *in vivo* using positron emission tomography (PET). Burns et al. [34] demonstrated that the selective, high-affinity inverse agonist for the CB_1_R, named [^18^F]MK-9470 had the potential to be a valuable tool for the *in vivo* study of CB_1_R biology and pharmacology. Several *in vivo* preclinical [35–41] and clinical studies [42–45] have used this compound successfully.

 We have recently reported higher levels of dopamine D_1_ and D_2_ receptors [46], both serotonin 5HT_1A_ receptor binding and mRNA expression [47], and GABA_A_ receptor binding [48] in adolescent rats (PND 39) compared to adults (PND 70), that is, in accordance with the regressive elimination of synapses and receptors that occurs during the transition from adolescence to adulthood [27]. In the present study, we have undertaken two objectives: first, to demonstrate the feasibility of imaging CB_1_R *in vivo* in adolescence and adulthood using small animal PET with [^18^F]MK-9470; second, to compare the level of expression/regional distribution of CB_1_R in adolescent and adult rats obtained *in vivo* with PET and *in vitro* with autoradiography using [^3^H]CP55,940. The aim was to test the hypothesis whether CB_1_R pruning occurs during the transition from adolescence to adulthood as it has been indicated for other neuroreceptor systems.

## 2. Materials and Methods

### 2.1. Radiochemical Synthesis of [^18^F]MK-9470

CB_1_R imaging was performed in all animals using the radioligand [^18^F]MK-9470 (N-[2-(3-cyanophenyl)-3-(4-(2-[^18^F]fluoroethoxy)phenyl)-1-methylpropyl]-2-(5-methyl-2-pyridyloxy)-2-methylpropanamide), a high specificity, high-affinity inverse agonist at the CB_1_R. The precursor for radiotracer synthesis and the authentic [^19^F]MK-9470 standard were obtained from MERCK Research labs (West Point, Pa, USA). Radiolabelling was performed using a two-step semiautomated procedure following the method outlined by Burns et al. [34] with some modifications. In the first step, 2-Bromo-1[^18^F]fluoroethane ([^18^F]BrFE) was synthesised using a Nuclear Interface FDG synthesizer (GE Medical System). ^18^F-Fluoroalkylation of the MK-9470 precursor was then manually carried out using Cs_2_CO_3_ as a base. An aliquot of [^18^F]BrFE was added, and [^18^F]MK-9470 was obtained in up to 8% overall yield (not corrected for decay) after high-performance liquid chromatography (HPLC) and Sep-Pak purification. [^18^F]MK-9470 product was confirmed by coinjection with the [^19^F]MK-9470 standard. The final product obtained had a radiochemical purity > 95% and specific activity averaging 6000 Ci/mmole (222 GBq/*μ*mole).

### 2.2. Animals

Male Wistar rats were obtained from the Animal Resource Centre Pty. Ltd (Perth, Australia) and were housed in polyethylene boxes with wire lids (489 × 343 × 240 mm) in groups of two-three per cage. All handling of animals and procedures was carried out in accordance with the guidelines established by the Animal Care and Ethics Committee at the Australian Nuclear Science and Technology Organisation (ANSTO). The animals were kept at a constant temperature of 22 ± 2°C on a 12–12 h light-dark cycle with lights on at 9 am and were handled during the seven days preceding the experiment. Food and water were freely available.

The adult cohort consisted of 6 rats with body weights ranging between 381 ± 22 g at 10 weeks of age (PND 70–72), and the adolescent cohort consisted of 6 rats with body weights ranging between 148 ± 22 g at 7 weeks of age (PND 35–37).

### 2.3. *In Vivo* PET/CT with [^18^F]MK-9470

#### 2.3.1. Acquisition and Reconstruction

Animals were fasted for at least 6 hours before the start of the experiment. PET imaging with [^18^F]MK-9470 was performed with a preclinical PET/CT Inveon (Siemens) system [49]. Anaesthesia was induced by exposing rats to 4% isoflurane in oxygen and then maintained by reducing the ratio to 1.5–2.5% for the duration of the studies. Isoflurane anesthesia has been shown not to have any significant effects on absolute [^18^F]MK-9470 binding as compared to control conditions [36]. The eyes were coated with a lubricating eye ointment (Allergan Inc., Ireland). Body temperature was maintained by a heating pad set at 38°C and monitored rectally. Heart rate (333.2 ± 25.9 beats/min), respiratory rate (41.6 ± 9.2 cycles/min), and saturation in oxygen (>95%) were measured with a pulse oximeter (Starr, Life Sciences Corp, USA). We also monitored the respiratory rate under the CT part of the scanner with a pressure sensor connected to a computer (Biovet, m2m imaging crop, USA). After anaesthesia and placing of the animal in the scanner with the help of laser guidance, a catheter was placed in a lateral tail vein of the rat and connected to an infusion pump (Harvard Apparatus, USA). A 60 min PET scan was started at the same time of the start of the one-minute injection of [^18^F]MK-9470 at a constant tracer mass (65.2 ± 1.5 pmoles). A 15 min CT scan was systematically performed after the PET scan. Activity volumes were reconstructed with an iterative reconstruction (OSEM/MAP) [50] including attenuation and scatter correction, achieving a reconstructed spatial resolution of 1.5 mm.

#### 2.3.2. Data Analysis

**Figure 1:**
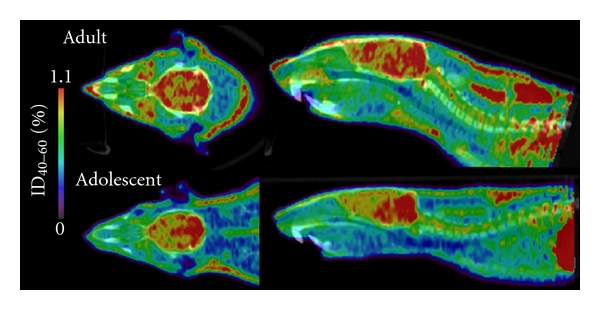
Typical *in vivo* PET/CT scan images of an adolescent (PND 35–37) and an adult (PND 70–72) Wistar rat in transversal (left) and sagittal (right) planes. For illustration purpose, the absolute binding intensity of [^18^F]MK-9470 to CB_1_R (%ID_40–60_) was increased (Anatomist/BrainVisa, V3.1.4, http://brainvisa.info/) in order to reflect the results expressed in estimated marginal means of %ID_40–60_, that is, a higher CB_1_R absolute binding in adults compared to adolescents.

**Figure 2:**
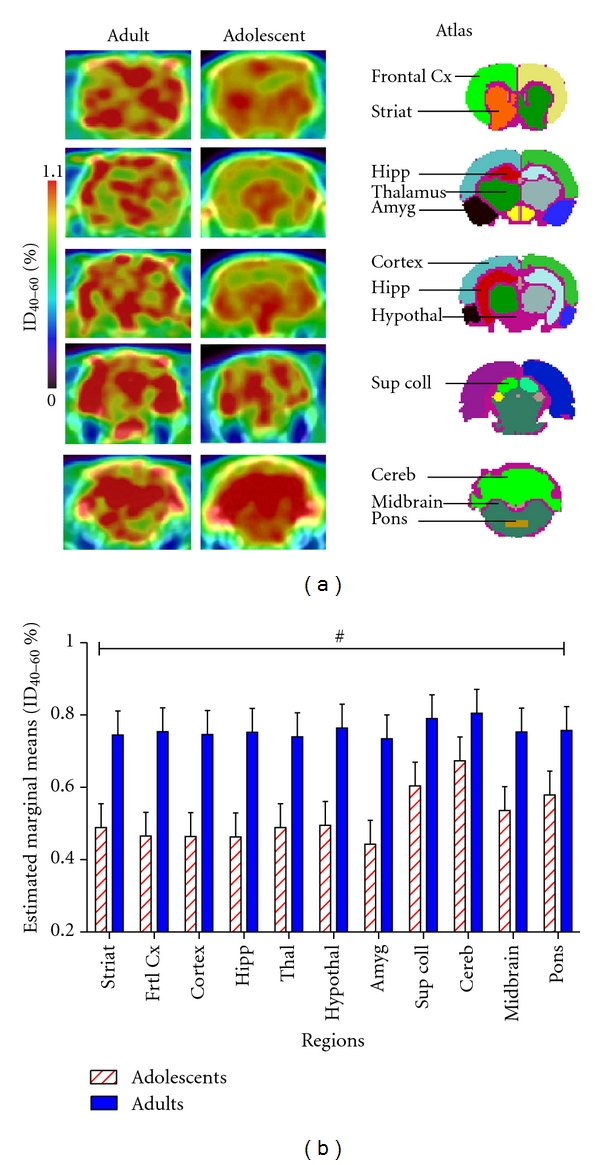
(a) *In vivo* PET/CT images of [^18^F]MK-9470 binding (%ID_40–60_ ± SEM) at 5 different coronal levels in the adolescent and the adult rat brain. The MRI-based atlas of the rat brain with 11 VOI is shown on the right side of the image. (b) Histograms presenting the adjusted absolute [^18^F]MK-9470 binding intensities (estimated marginal means of %ID_40–60_ ± SEM) in the adolescent compared to the adult cohort in 11 VOI. Two-way ANCOVA (age × region) controlling for weight was used to assess statistical significant differences in absolute [^18^F]MK-9470 binding between adulthood and adolescence. Statistical analysis revealed that there was a significant (^#^ *P* < 0.05 significant main effect) increase in CB_1_R absolute binding (44.4% calculated over 11 VOI) in adulthood compared to adolescence. Abbreviations: Striat: striatum; Frtl Cx: frontal cortex; Hipp: hippocampus; Thal: thalamus; Hypothal: hypothalamus; Amyg: amygdala; Sup Coll: superior colliculus; Cereb: cerebellum.

A previously developed magnetic-resonance-imaging- (MRI-)based rat brain atlas was coregistered to the PET volume, using the CT information of the skull (Anatomist/BrainVisa, V3.1.4, http://brainvisa.info/). In detail, all PET acquisitions (12 animals) were coregistered with their respective CT (see Figure 1). All CTs in the adolescent cohort were manually/visually coregistered to one adolescent CT (adolescence reference CT). The same methodology was used in the adult group. Finally, the “reference” CTs were manually/visually coregistered to the MRI-based rat brain atlas encompassing eleven volumes of interest (VOI) (Figure 2(a)). Transformation matrixes were then created from the MRI-based rat brain atlas to each PET image in each group.

Previous studies in rats with [^18^F]MK-9470 have used the last 20 min of a 60 min acquisition period (40 to 60 min) for quantification purposes [35–37]. In this study, we used percentage of injected dose (activity concentration (MBq/mL) divided by injected dose (MBq)) of the last 20 minutes of acquisition (%ID_40–60_) as absolute CB_1_R binding measure.

### 2.4. *In Vitro* Autoradiography with [^3^H]CP55,940

#### 2.4.1. Experiments

Twenty-four hours after *in vivo* imaging, the animals (6 adolescents and 5 adults) were euthanized, their brain was dissected, frozen in liquid nitrogen, and stored at −80°C. Coronal brain sections (16 *μ*m) were cut with a cryostat and thaw-mounted onto microscope slides.

[^3^H]CP55,940 autoradiography was carried out based on the method previously described in Dalton et al. [51]. All sections were processed simultaneously to minimize experimental variance. On the day of the experiment, sections were taken out of the −80°C freezer and allowed to come to room temperature for approximately 60 min or until dry. Sections were preincubated for 30 min at room temperature in 50 mM Tris-HCl (pH 7.4) containing 5% bovine serum albumin (BSA) in order to equilibrate the tissue to the assay conditions and remove any endogenous ligand. Radioligand binding was measured using single-point saturation analysis which provides a good estimate of receptor density. The Kd of rat brain CB_1_R has been evaluated at 5.2 nM [11]. In order to ensure saturation of CB_1_R, sections were then incubated for 2 h at room temperature in the same buffer as preincubation with the addition of 10 nM [^3^H]CP55,940 (specific activity 139.6 Ci/mmole, Perkin Elmer, USA). Nonspecific binding was determined by incubating adjacent sections in the presence of 10 *μ*M CP55,940. The concentration of [^3^H]CP55,940 was measured in 10 *μ*L aliquots taken from the incubation mixture. After the incubation, sections were washed for 1 h at 4°C in 50 mM Tris-HCl (pH 7.4) containing 1% BSA, and a second wash was then carried out for 3 h in the same buffer at 4°C. The third wash was in 50 mM Tris-HCl (pH 7.4) for 5 min at 4°C. Sections were then dipped briefly in ice cold distilled water and then dried. Dried sections were apposed to Kodak Biomax MR films, together with autoradiographic tritium standards ([^3^H] microscales from Amersham), in X-ray film cassettes. Films were developed after 35 days using Kodak GBX developer and fixed with Kodak GBX fixer.

#### 2.4.2. Data Analysis

Films were analysed using a computer-assisted image analysis system, Multianalyst, connected to a GS-690 Imaging Densitometer (Bio-Rad, USA). Eleven brain regions of interest (ROI) were manually drawn with the help of a stereotaxic atlas of the rat brain [52] and corresponded to the 11 VOI analysed *in vivo* (Figure 4). Quantification of receptor binding in each brain region was performed by measuring the average optical density in adjacent brain sections. Nonspecific binding was subtracted to total binding to give a value for specific binding. Optical density measurements for specific binding were then converted into fmoles of [^3^H]CP55,940 per mg of tissue equivalent (fmol/mg TE) according to the calibration curve obtained from the [^3^H]-labelled standards.

### 2.5. Statistical Analysis

Statistical tests were performed using PASW Statistics (Version 18.0.0) and Graphpad Prism (Version 5.04). Data were analysed for significant outliers (±2 SD), and none were detected. The Kolmogorov-Smirnov test was used to test normality of the data. Parametric tests were used in subsequent analysis since data were normally distributed. The mass and injected dose of [^18^F]MK-9470 between the adolescent and adult cohorts were compared using unpaired Student's *t*-tests. Pearson correlations were used to examine the relationship between %ID_40–60_ and body weight and between [^18^F]MK-9470 CB_1_R binding *in vivo* and [^3^H]CP55,940 CB_1_R binding *in vitro*. Analysis of covariance (ANCOVA) controlling for body weight was used to determine if there was an effect of age and/or region on CB_1_R absolute binding measured *in vivo*. *In vitro* data were analysed using two-way ANOVA (age × region) followed by least significant difference (LSD) tests. Significance was set at *P* ≤ 0.05.

## 3. Results

### 3.1. *In Vivo* PET with [^18^F]MK-9470

Adolescent rats showed the regional distribution that corresponds to the previously published regional distribution of CB_1_R [11, 53], but adult rats unexpectedly demonstrated a more uniform regional distribution of the PET radioligand (Figures 1 and 2). Cerebellum, striatum, cortical regions, and (moderately) hippocampus showed higher *in vivo* CB_1_R absolute binding compared to other brain regions. Regions known to have fewer CB_1_R like the thalamus and especially the brainstem (midbrain, pons) presented relatively high CB_1_R absolute binding *in vivo* (Figure 2).

Time-activity curves (expressed in %ID_40–60_) showed that [^18^F]MK-9470 entered the brain with a slow kinetic and reached a peak at approximately 20 min after-injection.

There were no statistically significant differences in the mass of [^18^F]MK-9470 injected between the adolescent and adult cohort (mean ± SEM: 64.8 ± 1.5 pM and 66.2 ± 2.9 pM, resp., *t*(10) = 0.73, *P* = 0.48). No statistical differences were found in the injected doses (ID) (*t*(10) = 0.56, *P* = 0.59) between the adolescents (8.22 ± 2.07 MBq) and the adults (7.02 ± 0.59 MBq). Animal weights were found to be significantly different (*t*(10) = 18.16, *P* < 0.0001) between adolescent (148 ± 9 g) and adult animals (382 ± 9 g), and Pearson's correlation showed that weight was strongly and negatively correlated to %ID_40–60_  (*r* = −0.921, *P* < 0.0001). Two-way ANCOVA (age × region) controlling for weight showed a significant main effect of age (*F*(1,109) = 4.95, *P* = 0.028) with adults having higher CB_1_R absolute binding compared to adolescents (+44.4% over 11 VOI) (Figure 2(b)). A significant effect of region was also found (*F*(10,109) = 2.41, *P* = 0.012). No interaction was observed between age and region (*F*(10,109) = 0.84, *P* = 0.59).  Table 1 presents CB_1_R absolute binding levels in adolescents and adults before (unadjusted values) and after controlling for animal body weight (adjusted values).

### 3.2. *In Vitro* [^3^H]CP55,940 Autoradiography

Two-way ANOVA (age × region) showed a statistically significant main effect of age (*F*(1,99) = 17.323, *P* < 0.0001) with the adults having higher CB_1_R binding than the adolescents (Figure 4). A significant main effect of region (*F*(10,99) = 140.1, *P* < 0.0001) was also found. No interaction between age and region (*F*(10,99) = 1.62, *P* = 0.113) was observed. The significant main effect of age was further analysed by LSD post hoc tests revealing that CB_1_R-specific binding was significantly higher in the adults compared to adolescents in the frontal cortex (+23.4%; *P* = 0.024), the cortex (+27.1%; *P* = 0.020), the hippocampus (+15.4%; 0.018), and the cerebellum (+15.2%, *P* = 0.002) (Table 2 and Figure 4).

### 3.3. Correlation between [^18^F]MK-9470 CB_1_R Binding *In Vivo* and [^3^H]CP55,940 CB_1_R Binding *In Vitro*

Correlations were not statistically significant between absolute CB_1_R binding evaluated with [^18^F]MK-9470 *in vivo* and specific CB_1_R binding calculated with [^3^H]CP55,940 *in vitro*, (*r* = 0.1816, *P* = 0.41).

## 4. Discussion

In the present study, we used two complementary techniques to examine potential developmental differences in CB_1_R binding in the brain of adolescent and adult rats. After controlling for body weight, CB_1_R absolute binding measured *in vivo* with PET and [^18^F]MK-9470 was significantly higher in the adult animals compared to adolescents over 11 brain regions. This finding was confirmed *in vitro* with autoradiography and [^3^H]CP55,940.

 Noteworthy, the percentage of increase observed in the adult compared to the adolescent cohort with the 2 complementary techniques was not of the same magnitude (44% *in vivo* versus 11% *in vitro* over the 11 regions of interest), and no significant correlation was found between the data obtained with the two techniques. The comparison between *in vitro* and *in vivo* results (no correlation) and the apparent discrepancies may relate to a number of factors.

Firstly, methodological issues of data analysis should be considered. The percentage of injected dose (% ID) that we used here gives an absolute index of binding *in vivo*. This means that it reflects specific and nonspecific binding in the brain, radioligand present in the brain blood circulation, and possible radioactive metabolites crossing the blood-brain barrier. We did not calculate standardised uptake values (absolute index normalising for weight) because it would have biased our results as our two groups had significantly different weight means. Also, the absence of a brain region devoid of CB_1_R prevented us from implementing a simplified reference tissue model. We chose to use an atlas-based analysis of our data, with predefined VOI, over a statistical parametric mapping approach because we wanted to compare the same regions with the *in vivo* and *in vitro* methodologies. To our knowledge, the metabolism of [^18^F]MK-9470 in the male (adult and adolescent) rat brain has not been assessed; therefore, the presence of active metabolites that cross the blood-brain barrier cannot be ruled out. Indeed, a metabolite is likely to cross the blood-brain barrier of adult female Wistar rats (Casteels et al., oral communication). Radiometabolites produced in adults (but not in adolescents) could potentially cross the blood brain barrier, affect the %ID we calculated, and in turn contribute to the uniform regional distribution of the PET radioligand we observed in adults compared to adolescents (Figures 1 and 2). Differences in radioligand present in the brain blood circulation (e.g., difference in blood flow) between the adolescent and adult cohorts that would have affected our measures cannot be ruled out either. To be in line with previous studies in rats with [^18^F]MK-9470 [35–37], we have used the last 20 min of a 60 min acquisition period (40 to 60 min) for quantification purposes. A recent study however indicated that the distribution volume (*V*_*T*_) of [^18^F]MK-9470 as quantitative outcome evaluated by full kinetic modelling was reasonably correlated with standardised uptake values between 60 and 80 min (Casteels et al., oral communication, 2011). Longer acquisitions periods (at least 80 min) in future studies using this radioligand would ensure that equilibrium is reached.

The second factor that could explain discrepancies between *in vitro* and *in vivo* results is the drug phenotype. Indeed, [^18^F]MK-9470 is an inverse agonist at CB_1_R [34], whereas [^3^H]CP55,940 is an agonist at both CB_1_R and CB_2_R [54, 55]. The concentration of CB_2_R in the rat brain is supposed to be small in comparison to CB_1_R [3, 56, 57]. Thus, [^3^H]CP55,940 binding in the brain will mainly reflect CB_1_R. Inverse agonists will preferentially bind to receptors uncoupled from their G-protein, whereas agonists will preferentially bind to receptors that are coupled to their G-protein [58]. This means that *in vivo* we would have preferentially bound CB_1_R uncoupled to their G-protein, whereas *in vitro* the G-protein-coupled ones would have been targeted. *In vitro* assays typically reflect all receptors that are available to bind to radioligand, whereas *in vivo*, only a subset of these receptors are available to bind to radioligand since some may be compartmentalised, some in a low affinity state and some occupied by endogenous ligand [59].

Finally, another factor affecting the comparison between *in vitro* and *in vivo* measures is the difference in concentration of the radioligand used. Theory of PET experiment is based upon the injection of a radioligand at tracer concentration that is not supposed to trigger any biological effect. In order to meet this requirement, the radioligand should not bind to more than 5–10% of the total receptors concentration (*B*_max⁡_) [59]. Based on previously reported *B*_max⁡_ in rat brain (0.5–1.1 pmol/mg prot) [11], we calculated that the mass of ligand needed to be approximately 0.1–0.7 nmoles. On the other hand, quantitative *in vitro* autoradiography studies need saturation of the available binding sites (at least 3 times greater than the *K*_d_). Thus, by saturating a different proportion of receptors *in vivo* and *in vitro*, differential outcomes must be cautiously interpreted.

 Our main results, showing an increase in CB_1_R in adults (PND 70–72) compared to adolescents (PND 35–37) *in vivo* and *in vitro*, are in accordance with *in vitro* studies that have looked at CB_1_R expression over time in development. Belue and collaborators [32] have found significant regional increases in the numbers of CB_1_R (*B*_max⁡_) in the developing rat brain (PND 0, 7, 14, 21, and 60) using *in vitro* autoradiography with [^3^H]CP55,940. Although CB_1_R density was not measured during adolescence, this study suggested that CB_1_R binding continuously increased until the maximum adult level was reached at PND 60. They observed that cortical regions (mainly posterior cortex) and hippocampus showed a statistically significant increase in binding between PND 21 and PND 60 [32]. According to the authors, the increase in CB_1_R could be an indication of either an increased differentiation of neurones into cells harbouring CB_1_R or an induction of the expression of CB_1_R in cells already differentiated. Another *in vitro* study using the same radioligand ([^3^H]CP55,940) showed that CB_1_Rs are transiently expressed in white matter areas during embryonic and early postnatal periods, progressively “shift” to their adult localization at PND 30, and increase between PND 30 and adulthood in the hippocampus, nucleus accumbens, and cerebral cortex [53]. In addition, Ellgren et al. [60] reported an increase in CB_1_R protein expression in the nucleus accumbens shell and no changes in prefrontal cortex between mid-(PND 38) and late-(PND 49) adolescence.

**Figure 3:**
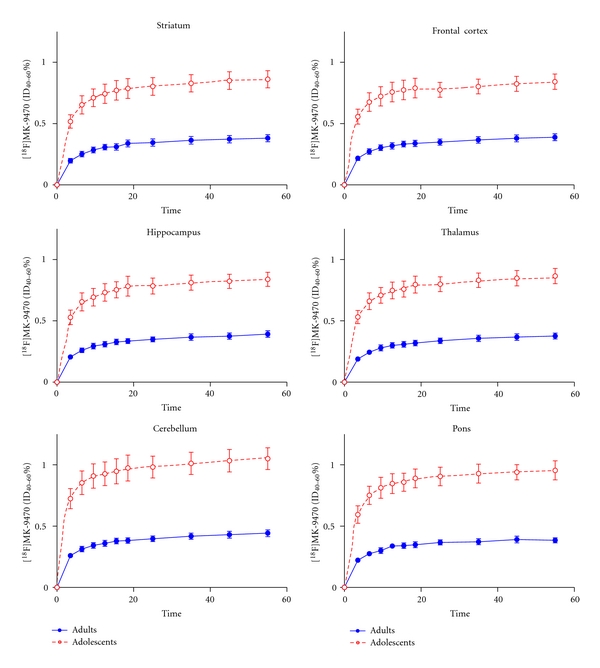
Time-activity kinetic curves of [^18^F]MK-9470 expressed in %ID_40–60_ ± SEM in 6 volumes of interest (VOI), in the adolescent (*n* = 6) (dotted line in red) and the adult (*n* = 6) (plain line in blue) cohort. Note that adolescents' kinetic curves appear higher compared to adults' kinetic curve because values are expressed as %ID_40–60_ not taking into account weight as covariate. Estimated marginal means of %ID_40–60_ were evaluated in the ANCOVA and showed higher [^18^F]MK-9470 absolute binding in adults compared to adolescents.

**Figure 4:**
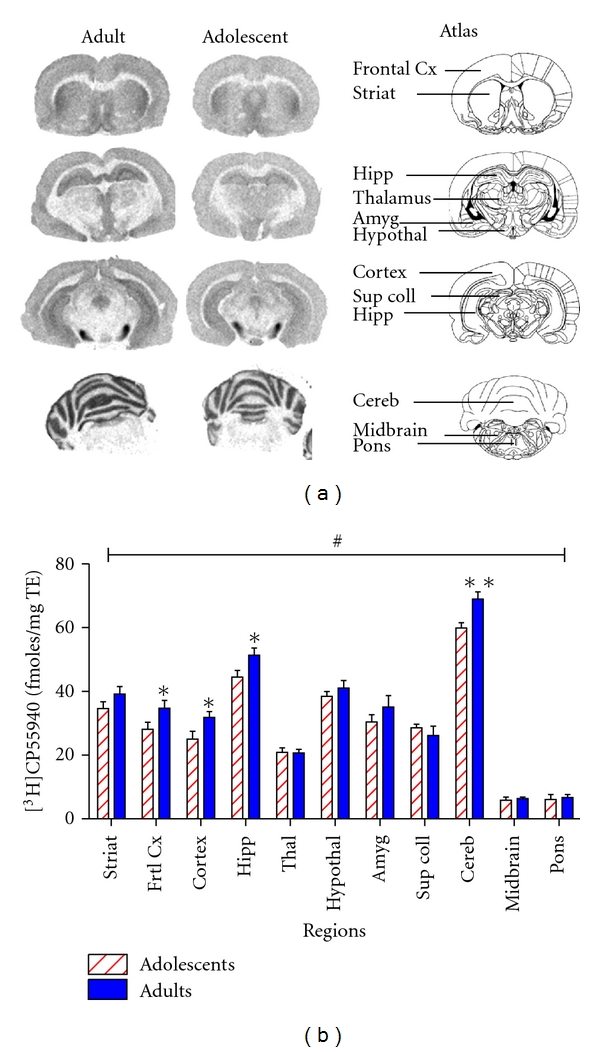
(a) *In vitro* autoradiographies of total [^18^F]MK-9470 binding intensity in coronal sections in an adolescent and an adult rat brain. The atlas of Paxinos and Watson [52] serves as a visual anatomical reference of the 11 brain regions analysed. (b) Histograms of the *in vitro* specific binding intensities of [^18^F]MK-9470 (fmoles/mg TE ± SEM) in the adolescent compared to the adult rat brain. Eleven regions of interest were analysed and assessed for statistical significant difference between adolescence and adulthood with two-way ANOVA (age × region) followed by LSD post hoc tests (*P < 0.05; **P < 0.01). Statistical analysis revealed a significant main effect (^#^ *P* < 0.05) of age with adults having higher CB_1_R densities than adolescents. The frontal cortex, the cortex, the hippocampus, and the cerebellum showed a statistically significant increase in adults compared to adolescence in the post hoc analysis. Abbreviations: Striat: striatum; Frtl Cx: frontal cortex; Hipp: hippocampus; Thal: thalamus; Hypothal: hypothalamus; Amyg: amygdala; Sup Coll: superior colliculus; Cereb: cerebellum.

In humans, an *in vitro* study found an increase in CB_1_R density between children/infant age (*n* = 5, 3 months to 8 years old) and adults (*n* = 5, 22 to 73 years old) in frontal cortex, hippocampus CA1 and DG, caudate putamen, globus pallidus, and cerebellum [61]. Interestingly, a recent PET study using [^18^F]MK-9470 found an increase in CB_1_R binding in the basal ganglia, lateral temporal cortex, and limbic system of aged female but not male humans [62]. Another PET study using [^11^C]OMAR in healthy males showed an age-associated decline in CB_1_R volume of distribution that was significant in globus pallidus only [63]. To allow comparison with other studies from our group [46, 51], we chose to evaluate the ontogeny of CB_1_R in adolescent and adult male rodents. Recent experiments have shown that female Wistar rats presented a high ~35–39% intersubject variability in CB_1_R binding evaluated as [^18^F]MK-9470 standard uptake values between 60 and 80 minutes (Casteels et al., oral communication, 2011). Intersubject variability in our study with males only was of 17% in the adult group and 18% in the adolescent group. Future *in vivo* animal studies looking at the ontogeny of CB_1_R in female rats as well as during aging would help in clarifying the relationships between gender, aging, and the endocannabinoid system (Figure 3).

In the mammalian brain, synapses and receptors within most regions are overproduced and eliminated during two phases of life. The first one occurs just before birth, after completion of the brain innervation, and witnesses the apoptosis (programmed cell death) of 50% of neurones in order to increase efficiency of synaptic transmission [64, 65]. The second one occurs during the periadolescence period with a tremendous overproduction of synapses and receptors followed by their progressive elimination or pruning [27]. This pattern of expression—overproduction followed by elimination—is shared among mammalian brains and part of a fundamental developmental strategy called “functional validation” [27]. Teicher et al. [66] reported an overproduction of D_1_ and D_2_ from PND 25 to 40 followed by a pruning to reach adulthood [66]. We have recently reported higher levels of dopamine D_1_ and D_2_ receptors [46], both serotonin 5HT_1A_ receptor binding and mRNA expression [47], and GABA_A_ receptor binding [48] in adolescent rats (PND 39) compared to adults (PND 70) that is in line with the regressive elimination of synapses and receptors that occurs during the transition from adolescence to adulthood. In contrast, the results of the present study indicate that CB_1_Rs are not undergoing a dramatic elimination between adolescence (PND 35–37) and adulthood (PND 70–72) and continue to increase, at least until PND 70–72. Our study does not rule out the possibility that the CB_1_Rs are undergoing pruning at a later developmental “aging” stage. Possible explanations for the observed upregulation in adult rats can be hypothesised. Since a homeostatic and modulatory role is attributed to endocannabinoids [57], the CB_1_R upregulation could be related to a compensation of functional losses in other monaminergic or GABAergic systems. In addition, changes in CB_1_R may reflect changes in endocannabinoid markers such as AEA and 2-AG. Limited information is available regarding endogenous cannabinoid ligands levels during the transition from adolescence to adulthood; however, a recent study has shown an increase of AEA but not 2-AG levels from early to late adolescence in the prefrontal cortex of the rats [60]. Studies looking at the developmental profile of endocannabinoid ligands in different brain regions and their correlations with CB_1_R levels would help in elucidating the developmental and morphogenic roles of this system during the transition from adolescence to adulthood.

## 5. Conclusion

The present study demonstrated the feasibility of imaging CB_1_R *in vivo* with PET and [^18^F]MK-9470 in adolescent and adult rats. Our results suggest that CB_1_Rs are increased during the transition from adolescence (PND 35–37) to adulthood (PND 70–72), a pattern that is opposite of most other neuroreceptor systems that have already started undergoing pruning during this time window. Availability of new radioligands such as [^18^F]MK-9470 in combination with PET would offer a unique opportunity to gain insights into the role of the endocannabinoid system during critical stages of development using longitudinal and within-subjects experimental designs and understand the consequences of its alterations after pharmacological challenges as well as in neurodevelopmental animal models of psychosis.

## Figures and Tables

**Table 1:** CB_1_ receptor *in vivo* binding levels ([^18^F]MK-9470) in adolescents and adults rats.

	Adolescents		Adults		% change (adjusted)
	Unadjusted	Adjusted	Unadjusted	Adjusted	
Striatum	0.86 ± 0.07	0.49 ± 0.07	0.38 ± 0.03	0.74 ± 0.07	50.3
Frontal cortex	0.83 ± 0.06	0.47 ± 0.07	0.38 ± 0.03	0.75 ± 0.07	59.8
Cortex	0.83 ± 0.07	0.47 ± 0.07	0.38 ± 0.03	0.74 ± 0.07	58.5
Hippocampus	0.83 ± 0.06	0.47 ± 0.07	0.38 ± 0.03	0.75 ± 0.07	59.9
Thalamus	0.86 ± 0.06	0.49 ± 0.07	0.37 ± 0.03	0.74 ± 0.07	49.1
Hypothalamus	0.86 ± 0.06	0.50 ± 0.07	0.40 ± 0.03	0.76 ± 0.07	52.0
Amygdala	0.81 ± 0.06	0.45 ± 0.07	0.36 ± 0.03	0.73 ± 0.07	63.1
Superior colliculus	0.97 ± 0.07	0.61 ± 0.07	0.42 ± 0.03	0.79 ± 0.07	29.2
Cerebellum	1.04 ± 0.09	0.68 ± 0.07	0.44 ± 0.03	0.80 ± 0.07	18.2
Midbrain	0.90 ± 0.06	0.54 ± 0.07	0.38 ± 0.02	0.75 ± 0.07	38.6
pons	0.95 ± 0.07	0.58 ± 0.07	0.39 ± 0.03	0.75 ± 0.07	29.0

Unadjusted values are mean %ID_40–60_ ± SEM; adjusted values are estimated marginal means %ID_40–60_ ± SEM.

Two-way ANCOVA controlling for weight was performed (*n* = 6 per group).

Covariates appearing in the ANCOVA model are evaluated at the following values: weight = 264.9142.

**Table 2:** CB_1_ receptor *in vitro* binding levels ([^3^H]CP55,940) in adolescents and adults rats.

	Adolescents	Adults	% change	*P* value
Striatum	34.62 ± 2.18	39.13 ± 2.38	13.0	0.117
Frontal cortex	28.12 ± 2.21	34.68 ± 2.51	23.4	**0.024**
Cortex	25.02 ± 2.49	31.81 ± 1.86	27.1	**0.020**
Hippocampus	44.51 ± 2.03	51.36 ± 2.26	15.4	**0.018**
Thalamus	20.90 ± 1.41	20.70 ± 1.16	−0.9	0.946
Hypothalamus	38.46 ± 1.53	41.01 ± 2.40	6.6	0.376
Amygdala	30.41 ± 2.29	35.09 ± 3.62	15.4	0.106
Superior colliculus	28.58 ± 1.12	26.16 ± 2.93	−8.5	0.400
Cerebellum	59.89 ± 1.66	68.98 ± 2.23	15.2	**0.002**
Midbrain	5.86 ± 0.96	6.35 ± 0.51	8.4	0.864
Pons	6.12 ± 1.50	6.70 ± 0.92	9.5	0.840

Two-way ANOVA followed by LSD post hoc test.

Data expressed as mean fmol/mg TE ± SEM; *n* = 6 per group.
